# Development and validation of an electronic frailty index in a national health maintenance organization

**DOI:** 10.18632/aging.206141

**Published:** 2024-10-24

**Authors:** Fabienne Hershkowitz Sikron, Rony Schenker, Yishay Koom, Galit Segal, Orit Shahar, Idit Wolf, Bawkat Mazengya, Maor Lewis, Irit Laxer, Dov Albukrek

**Affiliations:** 1 Meuhedet Health Maintenance Organization (HMO), Tel-Aviv, Israel; 2 The Joint-Eshel Organization, Jerusalem, Israel; 3 Department of Geriatrics, Israeli Ministry of Health, Jerusalem, Israel

**Keywords:** frailty, older people, electronic frailty index, electronic health record, health maintenance organization

## Abstract

Background: Frailty constitutes a major factor that puts the elderly at risk of health and functional deterioration.

Objectives: To develop and validate an Electronic Frailty Index based on electronic data routinely collected in the HMO.

Study design and setting: A retrospective cohort of the HMO members.

Participants: 120,986 patients, aged 65 years and over at the beginning of 2023.

Predictors: A cumulative frailty index including 36 medical, functional, and social deficits.

Outcomes: One-year all-cause mortality or hospitalization.

Statistical analysis: One-year hazard ratios were estimated for composite outcome of mortality or hospitalization using multivariable hierarchical Cox regression.

Results: The mean EFI score increased with the Social Security Nursing Benefit. Compared to fit patients, mild, moderate, and severe frailty patients had 2.07, 3.35, and 4.4-fold increased risks of mortality or hospitalization, after controlling for covariates.

Conclusions: The findings showed that the Electronic Frailty Index version we created is valid in predicting mortality or hospitalization. In addition, the Electronic Frailty Index converged with an independent measurement produced by National Social Security.

## INTRODUCTION

Advanced technology and improvements in medical services have resulted in an increased average life expectancy [1]. This achievement is accompanied by a challenge for us as a society to ensure that these added years in the lifespan will be healthy and functional for as long as possible.

Chronological age is an important determinant of health and survival among the elderly, but it appears that this is not the only factor, nor the most important one. We can easily see that individuals of the same chronological age can be very different from one another concerning their health status and functional ability. Frailty, a state of physiological vulnerability, constitutes a major factor that puts the elderly at risk of health deterioration and functional decline [2]. Research indicates that monitoring frailty offers a significant advantage compared to tracking age in predicting mortality and that it also outperforms chronological age as a predictor of disability [3].

There are two main approaches to measuring frailty in primary care. One method is the Fried’s frailty phenotype assessing only physical frailty through five criteria: unintentional weight loss; weakness or poor handgrip strength; self-reported exhaustion; slow walking speed; and low physical activity [4]. The second one is the Rockwood and Mitnitski’s cumulative deficit model, the approach we adopted in this article, assessing frailty based on the accumulation of physical, emotional, and social deficits [5]. Although some of the deficits are considered nonvital, such as hearing impairment or social vulnerability, the whole measure still predicts mortality [6]. The underlying explanation Rockwood and Mitnitski provide is that deficits in any complex system (such as the human being) make it more vulnerable, due to loss of interconnectedness of the parts which can create a multi-system failure. In other words, the coordination of the different systems is vital for life. A systematic review and meta-analysis based on 18 cohorts demonstrated that frailty measured by the FI is a significant predictor of mortality and even outperforms the frailty phenotype [7].

The electronic Frailty Index (EFI) developed by Clegg et al. [8] uses the cumulative deficit model as the theoretical framework but suggests an electronic version to operationalize the frailty concept. The EFI identifies frailty using routine data collected in primary care and automatically saved in clinical databases. As frailty indicators are stored in electronic health records (EHRs), additional resources are not required to produce the index. The EFI identifies 36 deficits and classifies individuals as ‘fit’ or exhibiting frailty in the ‘mild’, ‘moderate’, or ‘severe’ range. The EFI has demonstrated robust predictive validity for outcomes of hospitalization, residential aged-care admission, and mortality [8].

Since the operative definitions of such an index are specific to the country in which it was developed [9, 10], different versions of the EFI in primary care settings were developed in different countries, including, to name just a few, the US [11, 12], Canada [13], Australia [14], China [15], Japan [16], Spain [17], Sweden [18], Italy [19] and in other parts of the United Kingdom such as Wales [20] and Scotland [21]. The EFI was used in primary care settings but also in other settings such as hospitalized older adults [18, 22–24], pre-operative [25] and postsurgical patients [26], individuals with chronic kidney disease [27], with pulmonary hypertension [28] and with heart failure [29], residential aged care homes [30], and hospitalized COVID-19 patients [22].

Alongside many studies that focused on the EFI as a valid predictor of mortality or hospitalization outcomes [17, 22, 26, 28], other studies focused on convergent validities of the EFI with other frailty measures. Those studies showed, among other findings, high correlation with the Edmonton Frail Scale [31], Fried’s frailty scale [32], clinical measures of frailty such as ADL and IADL [33], frailty codes collected on an ongoing basis at the primary care clinic [34], and comprehensive geriatric assessment (CGA) [13, 35].

Studies that specifically investigated the use of EFI in the context of community primary care found a relationship between frailty and the number of community referrals per patient [36], a negative correlation with socioeconomic status [37], higher EFI scores in areas with higher levels of deprivation [21], differences in the prevalence of frailty between ethnic groups [38], a positive correlation with body mass index [39] and relation between EFI and polypharmacy due to the adverse actions from the drugs [40].

Despite the large number of studies that validated the EFI or looked for an association between EFI and other characteristics, the literature still states that further research is needed to develop and validate frailty assessment tools based on EHRs in other parts of the world [41, 9]. It is necessary for countries that want to use the EFI to adjust it in terms of definitions or codes according to their specific needs and database systems. Mitnitski and Rockwood, recommended that it includes at least 30 items; but apart from this criterion, they suggest that even if each adaptation includes a slightly different list of deficits, as well as a different number of items, the EFI is sufficiently robust and not sensitive to the choice of specific items.

The objective of our study was to develop and validate a version of the EFI adapted to our needs and to the EHR data routinely collected in the Israeli Meuhedet Health Maintenance Organization (HMO). At the method level, a detailed description of how to produce the index may help health organizations interested in building their own index. At the level of findings, this real-world setting data, based on a large population, may contribute to other countries with similar characteristics in terms of cultural and racial diversity as a source of comparison.

## RESULTS

### Participants

The cohort included all the 120,986 patients of the Meuhedet HMO aged 65 and over, 54.3% of whom were females (See Table 1). The mean continuous age was 73.9 (SD = 7.0), with a median of 72, a range from 65 to 106 and an interquartile range of 68 to 78. More than half belonged to the middle social level. The mean of the continuous Charlson comorbidity index was 2.1 (SD = 2.2), with a median of 2, a range from 0 to 23, and an interquartile range of 0 to 3.

**Table 1:** Baseline characteristics, MEFI medians and outcome rates.

**Characteristic**	**Frequencies**	**MEFI median (interquartile)**	*P*-value -MEFI differences^*^	**Outcome rate (%)**	*P*-value -outcome differences^**^
ALL	100%	0.17 (0.11–0.22)		18.40%	
Frailty cat					*P* < .0001
Fit	37%	0.08 (0.06–0.11)		7.90%	
Mild	40%	0.17 (0.14–0.19)		18.40%	
Moderate	17%	0.28 (0.25–0.31)		32.50%	
Severe	6%	0.39 (0.36–0.39)		45.20%	
Gender			*P* < .0001^*^		*P* < .0001
Male	45.70%	0.14 (0.08–0.22)		20.70%	
Female	54.30%	0.17 (0.11–0.22)		16.40%	
Age groups			*P* < .0001^**^		*P* < .0001
65–74	60.50%	0.14 (0.08–0.19)		14.10%	
75–84	29.90%	0.19 (0.14–0.25)		22.20%	
85+	9.60%	0.25 (0.19–0.33)		33.90%	
SES			*P* < .0001^**^		*P* < .0001
Low	25.40%	0.17 (0.11–0.25)		20.00%	
Middle	54.10%	0.17 (0.11–0.22)		18.30%	
High	20.50%	0.14 (0.08–0.19)		16.30%	
CCI groups			*P* < .0001^**^		*P* < .0001
0	31.20%	0.08 (0.06–0.14)		9.40%	
1–2	35.00%	0.14 (0.11–0.19)		16.60%	
3–5	26.30%	0.22 (0.17–0.28)		24.70%	
6+	7.50%	0.31 (0.25–0.36)		42.20%	

^*^Mann-Whitney *U*-Test; Kruskal-Wallis. ^**^Chi-square.

### Descriptive statistics of the MEFI

**Figure 1:**
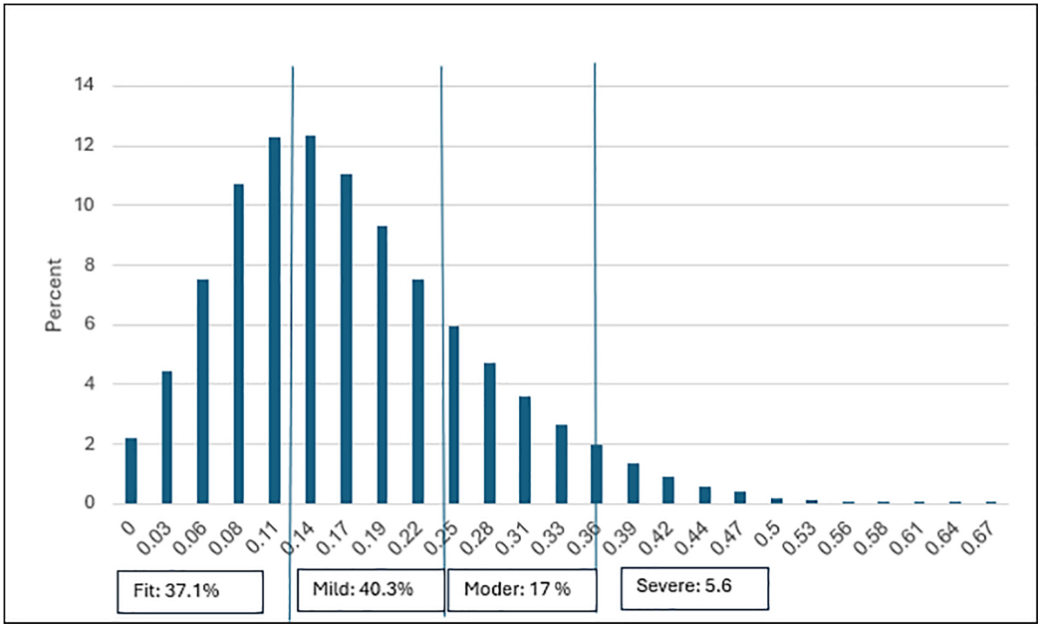
MEFI score bar chart in percent.

The MEFI (Meuhedet Electronic Frailty Index) distribution was right-skewed (see Figure 1). The mean MEFI score was 0.17 (SD 0.10), with a median of 0.17. Regarding the number of different deficits, the mean was 6.1 (SD 3.54), with a median of 6 different deficits, ranging from 0 to 24 different deficits; the interquartile range was 4–8. Among the cohort, 2639 patients (2.2%) had no deficits at all, mostly at the younger end of the range, 28% of them without any registered healthcare contact in the year preceding the follow-up (2022).

The MEFI score was slightly but significantly (*p* < .001) higher for females (see Table 1); it increased with age groups (*p* < .001) and decreased with socio-economic status (*p* < .001). The average level of frailty increased with age at a rate of 4% per year.

At the categorical variable level, the prevalence for fit, mild frailty, moderate frailty, and severe frailty categories were 34%, 42%, 18%, and 6%, respectively.

### Descriptive statistics of the outcome

Out of 120,986 patients, 17.9% were hospitalized at least once and 2.3% died during 2023. Of those who were hospitalized, 10.1% eventually died, compared to 0.6% of those who were not hospitalized. Overall, 18.4% experienced an adverse outcome of hospitalization or death by 2023. The adverse outcome rate increased with frailty from 7.9% for the fit and up to 45.2% for the severe frailty group (see Table 1). The rate also increased with age from 14.1% for the 65–74 age-old group to 33.9% for the oldest group, was higher among males, and decreased with socio-economic status from 20.0% for the lowest socio-economic group to 16.3% for the highest group. The adverse outcome rate increased with CCI from 9.4% for the group with 0 comorbidities on the CCI and up to 42.2% for the highest CCI group.

### Convergent validity

**Figure 2:**
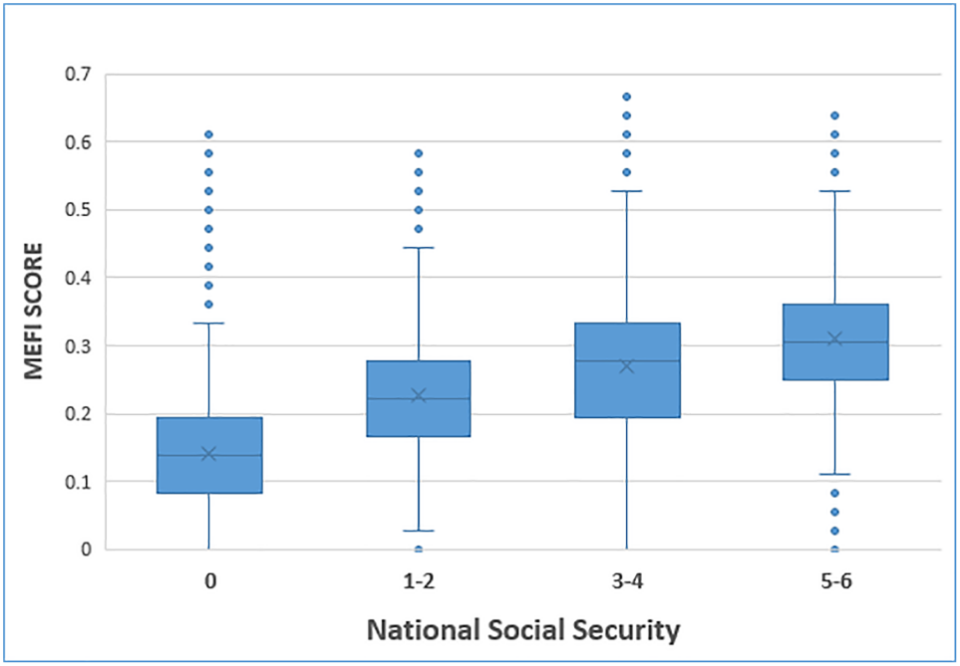
Boxplot of MEFI grouped by Social Security benefits.

Most of the patients didn’t receive any benefit at all (78.4%), 7.9% were at the lowest range of the score (1–2), 8.6% were at the middle range (3–4), and 5.0% were at the highest range (5–6). The median MEFI score increased with the benefits and was 0.14, 0.22, 0.28, and 0.31 for the group without benefits, and the lower, middle and higher groups, respectively. The increase between each step was significant (Kruskal Wallis and pairwise comparisons: *p* < .001). (See Figure 2).

### Predictive validity

**Figure 3:**
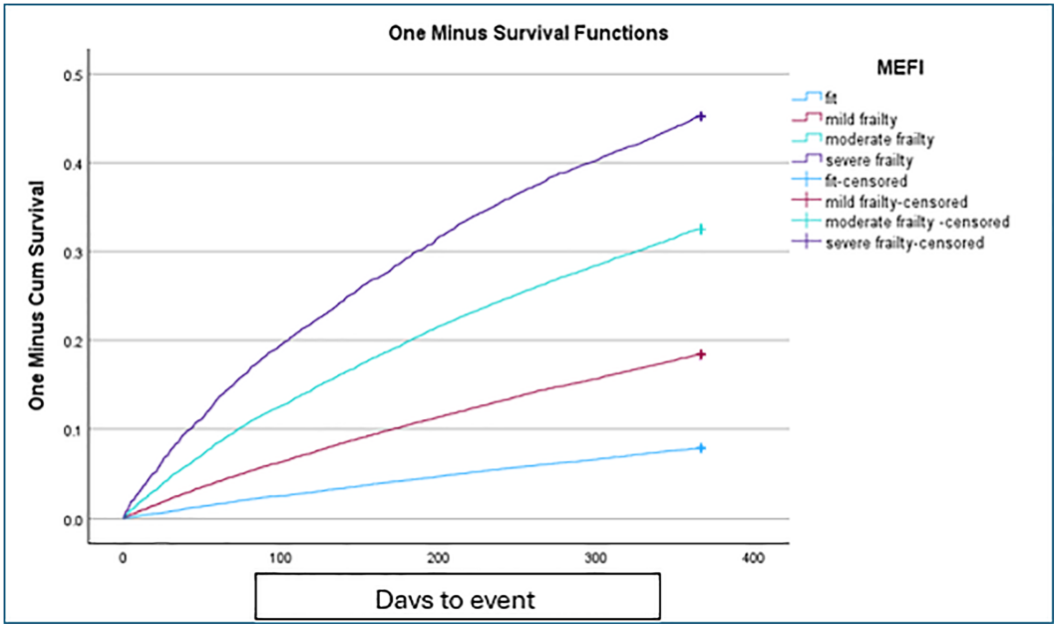
One-year Kaplan-Meiers survival curve for hospitalization or mortality outcome, by MEFI categories.

Figure 3 shows Kaplan–Meier estimates of hospitalization or mortality. As expected, we observed a significant increase in adverse outcome with increasing MEFI categories (Log-Rank (Mantel-Cox) = 10161 <.001). The AUC for hospitalization or mortality vs. continuous MEFI was 70.6% (95% confidence interval (CI) = 0.703–0.710). For fit, mild and moderate upper borders cutoffs the sensitivity and specificity were 0.84 and 0.58; 0.13 and 0.18; and 0.09 and 0.02, respectively. The MEFI significantly outperformed the CCI with a greater discriminative ability for a one-year outcome, and an AUC difference of 0.048 (95% confidence interval (CI) = 0.045–0.052). Spearman’s rho correlation between MEFI and CCI was 0.64 (*p* < .01).

A Cox regression of MEFI adjusted for gender, age groups and socio-economic status showed that compared to the MEFI reference category “fit”, the mild, moderate, and severe frailty categories were significantly more at risk of hospitalization or mortality (aHR = 2.40, 4.40 and 6.52, respectively, *p* < .001). A hierarchical multivariable Cox regression model, adjusted for gender, age groups, socio-economic status, and CCI categories showed that adding MEFI at block 3 achieved a significant improvement in fit relative to the previous block including only demographic and CCI predictors (X2(3) = 2584, *p* < .001) (see Table 2). In this final model, when compared to the MEFI reference category “fit”, the mild, moderate, and severe frailty categories were significantly more at risk of hospitalization or mortality (aHR = 2.07, 3.35 and 4.40, respectively, *p* < .001). All the other predictors in the adjusted model were significant as well, holding all the other covariates constant. Female gender was significantly associated with a 25% decrease in adverse outcome (aHR = 0.75; 95% CI: 0.73–0.77, *p* < .001). The risk of adverse outcome increased by 14% and 45% for those aged 75–84 and 85+, respectively, compared with those aged 65–75 (*p* < .001). The risk of adverse outcome decreased by 7% and 13% for those with middle and high socio-economic status, respectively, compared with low status. Patients with low (1–2), middle (3–5) and high CCI (6+), compared to patients with CCI 0, had 1.26, 1.38, and 1.99-fold increased risks of adverse outcome during the 1-year follow-up. Moreover, the pseudo-R2 estimates representing the goodness of fit of the model increased significantly between the levels of the hierarchical regression. We also performed an additional COX regression stratified by CCI as another way to isolate the effect of CCI from the effect of MEFI and we still found significant effects at each layer.

**Table 2:** Results of hierarchical Cox regression analysis for hospitalization or mortality outcome^**^.

	**Model 1**		**Model 2**		**Model 3**	
**Predictor**	**B**	**aHR**	**B**	**aHR**	**B**	**aHR**
**Female vs. male**	−0.30	0.74	−0.19	0.83	−0.28	0.75
**Age 65–74**
75–84	0.51	1.67	0.35	1.41	0.13	1.14
85+	1.02	2.78	0.75	2.11	0.37	1.45
**SES: low**
Middle	−0.12	0.89	−0.10	0.91	−0.08	0.92
High	−0.26	0.77	−0.20	0.82	−0.14	0.87
**CCI: 0**
1–2			0.54	1.71	0.23	1.26
3–5			0.92	2.50	0.32	1.38
6+			1.51	4.52	0.69	1.99
**MEFI: fit**
Mild frailty					0.73	**2.07**
Moderate frailty					1.21	**3.35**
Severe frailty					1.48	**4.40**
Chi-square	3773, df = 5, *p* < .001		8863, df =8, *p* <.001		11793, df = 11, *p* <.001	

^**^All the aHR were significant at *p* < .001.

### Sensitivity analysis

#### Missing values

Excluding 2816 patients (2.3%) without any contact with our medical staff the year before (2022) had almost no effect on the regression results (aHR = 2.06, 3.36, and 4.42 when comparing mild, moderate, and severe frailty to fit patients, respectively).

#### Stratification by age

When performing the same analysis stratified by age, the AUC for age groups 65–74, 75–84 and 85+ was still significant for each layer apart (AUC 0.70 (95% CI: 0.69–0.71); AUC 0.67 (95% CI: 0.66–0.68); AUC 0.62 (95% CI: 0.61–0.63), respectively). Moreover, the MEFI still significantly outperformed CCI for age 65–74 and 75–84 with an AUC difference of 0.049 (95% CI: 0.044–0.054) and 0.031 (95% CI: 0.025–0.037), respectively (ROC curve). The adjusted hazard ratios for adverse outcome were also significant at each layer apart (Cox regression), with the highest effect found among those aged 65–74 (aHR = 2.18, 3.82, and 5.63 when comparing mild, moderate, and severe frailty to fit patients, respectively).

## DISCUSSION

This work aimed to build an electronic frailty index and validate it. The combined prevalence of moderate and severe frailty in our electronic frailty index was quite similar to the prevalence found in Clegg’s cohort (20% and 24%, respectively), and even this difference can be explained by the fact that they limited the age of the cohort to 95 and we didn’t. The findings showed that the MEFI converged with nursing benefits granted, an independent measure performed by the National Social Security. The findings also showed that the MEFI version we created is valid in predicting mortality or hospitalization and had better predictive accuracy compared to CCI. The outperformance of MEFI suggests that aspects of health beyond chronic diseases, such as depression or hearing impairment, have also a decisive effect on those outcomes. This fact supports Rockwood’s approach claiming that deficits in any complex system make it more vulnerable, due to the loss of interconnectedness of the parts. It can be compared to a mosaic face painting where one of the parts is missing, the whole painting will be damaged, no matter which part is missing. In line with this approach, it was shown that interventions targeting risk factors such as sleep deprivation, and visual or hearing impairment may reduce delirium episodes in hospitalized older patients [42].

As a health maintenance organization, our mandate is to help our patients live longer and better. Using the MEFI as part of the routine primary care in our HMO may help us achieve this goal. Although the use of an electronic frailty index is a kind of shortcut that may be less accurate than frontal clinical assessment, mostly because of false-positive bias, using MEFI may be the first step before providing the most appropriate clinical care [43]. Moreover, in the case of intervention implementation, population segmentation used to identify the target group is very useful and the only risk with a false-positive assessment will be that the intervention will also be offered to some people that doesn’t need it so much. The classification of patients according to their level of frailty allows us to adjust prevention programs and focus our limited resources on the right action for the right person. For example, a program we developed for those released from hospitals is provided only to mild frailty patients, assuming that in this population it is possible to achieve a higher impact. From the literature, a study showed that people with the highest risk of death have a distinctive EFI trajectory in the last 12 months of life, with a rapid initial rise followed by a plateau [44]. This population can be identified using MEFI and can be integrated earlier into a palliative care program. It is known that frailty increases with age, affecting 10% of adults aged 50–64 and 43.7% of adults aged ≥65 [45] but the mean time spent in each frailty category before moving to a higher degree of frailty decreases with age, emphasizing the need to intervene on time [46]. Applying intervention programs before deterioration may keep the patient alive or prevent the next hospitalization. Hospitalization is the result of previous deterioration, but it is also a cause of future deterioration; therefore, an index that predicts future hospitalization is greatly needed. In addition, hospitalization constitutes the heaviest cost component for an HMO, far above the cost of clinic visits or medications [47]. Increased care for a person prone to deterioration may prevent hospitalization, thereby benefitting the patient while realizing significant HMO savings – which can be used to further finance the prevention activity. In addition to using the frailty index to identify populations at risk, it will be valuable to use the MEFI as an outcome measure in the evaluation of interventions, by comparing frailty before and after the interventions, compared with a control group [48].

The frailty index is not only a tool for managing populations and regulating the allocation of resources, but it is also clinically valuable at the individual level. England encourages a proactive approach and provides every primary-care physician with an automatic risk stratification tool based on the EFI [49]. We aim to develop a similar system in Meuhedet.

### Limitations

Building our customized frailty index required us to select deficits from a lot of options, and then to decide how to operatively define them and calculate the summarizing score. The multiple decisions made during the process were not unequivocal, despite the input of many experts; therefore, it is advisable to periodically review the algorithm and optimize it. Despite the above, it seems that the operative decisions are flexible, as Rockwood himself, who developed this theoretical framework, stated [50]:

“Whereas it is understandable to be concerned about the specific nature of the variables that might be included in the frailty index, our experience suggests that, when some sufficiently large number (roughly, about 40) variables are considered, the variables can be selected at random, and still yield comparable results of the risks of adverse outcomes”.

Another limitation is that people who have not been in contact with any health professionals in the HMO the year before will not have any functional disabilities recorded and will be considered patients who don’t suffer from any functional disabilities. This decision assumes that people with an acute medical problem will end up seeing a clinician sometime, somewhere. This assumption is especially true in Israel where there is a state health law that allows access to primary care physicians at no cost, against a low quarterly fee. Thanks to this method and thanks to the proactive outreach activities performed mostly by Meuhedet’s nurses, only 2.3% didn’t visit any health professionals the year before. A sensitivity analysis that did not include them showed no change in the findings.

## CONCLUSIONS

The MEFI has been proven to be valid and is already helping us to stratify our patients, adjust intervention programs adapted to their frailty status, and evaluate the effectiveness of those programs. In the future, the MEFI will hopefully be installed on the doctor’s computer as an automatic risk stratification tool.

## MATERIALS AND METHODS

We followed the equator STROBE (Strengthening the Reporting of Observational Studies in Epidemiology) reporting guideline [51].

### Study design

A retrospective population-based cohort study.

### Setting

The Meuhedet HMO is Israel’s third largest integrated healthcare service provider, serving over 1.3 million patients nationwide of all ages. Patient’s medical data are stored in a comprehensive data warehouse that combines hospital and community medical records, imaging and laboratory results, and pharmaceutical records. Patient-level data are maintained by Meuhedet from an operational database including socio-demographic data, and comprehensive clinical information such as coexisting chronic illnesses, community-care visits, medications, and results of laboratory tests. The data were extracted from Meuhedet EHR systems.

### Eligibility criteria

The cohort included all the 120,986 Meuhedet members aged 65 years and over in January 2023, including those who died during the year 2023 but not including those who left the HMO in the meantime.

### Follow-up period

The cohort was followed for one year (January–December 2023) for all-cause mortality or hospitalization.

### Data sources and measurement of the MEFI

The initial list of items included in the MEFI was mainly based on Clegg’s deficits [8] to which we added some Orkaby’s deficits because we believed in their importance, such as anxiety, depression, and dementia [11]. The list was discussed among a group of experts in the field of geriatric health. One of the criteria for determining the composition of the list was that the items would apply to all aspects of health: diseases, functioning, social interaction, and psychological health. Further, we conducted brainstorming meetings with therapists from a wide range of sectors, including geriatrics, family medicine, nursing, physiotherapy, occupational therapy, speech therapy, dietetics, pharmaceuticals, and social work. The purpose of the brainstorming was to determine in which of the sectors the deficits appear, and what specific codes should identify them. For example, we will know that a patient has problems related to instability or falling if the individual’s doctor visit was marked by a relevant International Classification of Diseases (ICD) code, such as fracture, in the clinic’s electronic file; or if the patient told a nurse of a fall during a routine checkup, and subsequently performed an ‘up and go’ test; or if a physiotherapist recorded that the patient was given exercises to improve stability. The look-back period for chronic diseases was from the age of 55, and the look-back period for non-disease deficits (such as functional deficits) was reduced to one year, a short range that corresponds to most EFI developed since then. The weight was the same for all the deficits, one point, conforming to Clegg's definition. If a deficit did not appear anywhere in the electronic record, it was recorded as 0 points. The assumption was that a patient who has a problem would usually see at least one of the HMO clinicians, either on his initiative or due to the staff’s proactive ongoing outreach activities. Only 2.3% of the elderly didn’t have any registered healthcare contact in the year preceding the follow-up (2022), and a sensitivity analysis was performed without them. The final list included 36 deficits with a prevalence of at least around 1% (not too rare) but below 80% (not saturated) [52]. They included chronic conditions, physical limitations, cognitive deficits, and general health, in line with the recommendations for constructing the EFI [5], see Table 3. The Frailty Index score was calculated as a sum of all points divided by the number of deficits (36).

**Table 3:** List of 36 deficits included in the MEFI: coding, look-back period and frequency in 2023.

**Deficits**	**Look-back period**	**Therapists and doctors’ ICD-9 codes**	**Frequ.2023**
Activity Limitation	12 months	Nurse, Occupational Therapy	1.2%
Anaemia and Haematinic Deficiency	12 months	280–285	8.4%
Anxiety	Chronic: from age 55; Visit diag: 12 months	Chronic anxiety 293.84, 300.0–300.1, 309.24, 309.28.	9.8%
Arthritis	From age 55	274, 446.5, 710.9, 714.0–714.2, 714.4, 714.89, 714.9, 715, 716.1–716.3, 716.5–716.6, 716.8–716.9, 725	55.4%
Atrial Fibrillation	From age 55	427.3, Z37.34	12.3%
Cancer (any except basal cell skin cancer)	From age 55	140–165, 170–172, 174–179, 180–209	21.2%
Cerebrovascular Disease	From age 55	362.34, 433–435, 436., 437.0–437.1, 438.0–438.5, 438.81–438.82, 438.89, 438.9, v12.54	18.4%
Chronic Kidney Disease	From age 55	250.4, 403.00, 403.10, 403.90, 404.00, 404.01, 404.10–404.11, 404.90–404.91, 582, 585–588. Dialysis 403.01, 403.11, 403.91, 404.02–404.03, 404.12–404.13, 404.92–404.93, V42.0, V56.0, V45.1, E879.1, 39.93, 39.95, 54.98	14.3%
Coronary Artery Disease	From age 55	410–412, 414, 429.2, 429.5, 429.7, V45.82, 00.66, 36.01–36.02, 36.04–36.05, 36.10–36.17, 36.19	22.4%
Dementias	From age 55	290.0–290.4, 291.1–291.2, 293.0–293.1, 294.8–294.9, 331.0, 331.10–331.11, 331.82–331.83, 331.92, 333.4, 438.0, 780.09, 780.93, 799.5	7.3%
Depression	Chronic: from age 55; Visit diag: 12 months	Chronic depression 296, 298.0, 309.0–309.1, 311	11.2%
Diabetes	From age 55	250.00, 250.02, 250.10, 250.12, 250.20, 250.22, 250.30, 250.32, 250.40, 250.42, 250.50, 250.52, 250.60, 250.62, 250.70, 250.72, 250.80, 250.82, 250.90, 250.92	32.8%
Dizziness/Vertigo	12 months	Medication (‘N07CA’), Speech Therapy, Physiotherapy	0.9%
Fall/ fall-related injuries (hip/skull fractures, subdural hematoma)	12 months	430, 733.14, 733.96, 800–801, 803, 835, 852, 880, 81.4, 81.51, 81.59, V43.6, E880, E884.2-E884.9, E885.9, E887, E888, Physiotherapy, Nurse, Up&Go Test	3.6%
Fatigue	12 months	780.7, 780.71, 780.79	3.2%
Gait Abnormality	12 months	719.7, 781.2–781.4	9.8%
Gastro-intestinal Disease	From age 55	531–534, 570–571, 572.2, 572.3, 572.8, 573	22.2%
Hearing Impairment	12 months	388.0–388.2, 389, V41.2, V53.2, 95.48, 95.49, Speech Therapy, Nurse	11.2%
Heart Failure	From age 55	428, 402.01, 402.11, 402.91, 404.01, 404.03, 404.11, 404.13, 404.91, 404.93	9.7%
Housebound	Last updated status	Nurse anamnesis, Home Care Unit, National Social Security	6.5%
Hypertension	From age 55	401–405	73.0%
Lung Disease	From age 55	490–496, 510	24.9%
Memory and Cognitive Problems	12 months	Occupational Therapy, Medication (‘N06D’), Nurse, Mini-Cog Test	24.2%
Muscular Wasting	12 months	307.1, 728.2, 728.87, 783.0, 799.3–799.4	9.2%
Osteoporosis	From age 55	733.00, 733.01, 733.02, 733.03, 733.09, 733.1, 733.10, 733.13	4.6%
Parkinson’s Disease	From age 55	332, 333.1	6.2%
Peripheral Neuropathy	From age 55	250.60, 250.62, 337.00, 337.09, 337.1, 356.4, 356.8, 357.1–357.7	0.8%
Peripheral Vascular Disease	12 months	250.70, 250.72, 440–444, 447, 451–453, 557	23.8%
Polypharmacy	12 months	8+ drugs	72.0%
Requires Care	12 months	Nurse	1.0%
Sleep Disturbance	12 months	Medication (‘N05CD09’, ‘N05CF01’, ‘N05CF02’, ‘N05CH01’), Nurse	2.3%
Social Vulnerability	If holocaust or poverty: last update; if therapist diag: 12 months	Nurse, Occupational Therapy, Holocaust survivor, Poverty	27.6%
Thyroid Disease	From age 55	242, 244–245, 246.0, 246.3–246.9	21.1%
Urinary Incontinence	Chronic diag: from age 55; visit diag: 12 months	625.6, 787.6, 788.3, 788.91, Medication (‘G04BD’)	10.0%
Vision Comorbidity	Blindness: from age 55; else 12 months	362.50–362.53, 365.05–365.13, 365.2–365.7, 365.81–365.82, 365.89, 365.9, 368.30–368.31, 368.4, 368.60, 368.62–368.69, 368.7, 368.8–368.9, 369 (blindness)	7.3%
Weight Loss in the past year	12 months	783.2 or Dietician	2.7%

The score continuum was then divided into categories of ‘fit’, ‘mild frailty’, ‘moderate frailty’, and ‘severe frailty’, according to Clegg’s cutoff points [8], to increase comparability with other studies [30]. Specifically, MEFI scores of 0–0.12 were defined as ‘fit’; >0.12–0.24 as having ‘mild frailty’; >0.24–0.36 as ‘moderate frailty’; and >0.36 as ‘severe frailty’. The whole process was accompanied by a steering committee consisting of frailty experts representing the Meuhedet HMO, the Joint-Eshel NGO, and the Geriatrics Department of the Israeli Ministry of Health.

### Predictors, potential confounders, and effect modifiers

Age groups: the age was divided into three categories: young–old (65–74), middle-old (75–84), and oldest-old (85+). These categories are based on biological aspects and the age distribution in our HMO.

Gender: males and females, as recorded in the electronic health record.

Socio-economic status (SES): derived from the individual’s home address and based on characteristics that are routinely collected by the Central Bureau of Statistics, ranging from 1 to 10. For this study, SES levels were grouped in the way we usually divide, into three levels: 1–4 low, 5–7 medium, and 8–10 high.

CCI: the Charlson Comorbidity Index (CCI) assesses comorbidity levels by considering both the number and severity of 17 pre-defined comorbid conditions [53]. The higher the score the higher the predicted mortality rate. CCI was categorized into 4 grades: no comorbidity (0), mild (1–2), moderate (3–5), and severe (6+). Five comorbidities out of 19 were common to both CCI and MEFI.

Nursing benefit: the nursing benefits are awarded by Israel’s National Social Security. National Social Security emphasizes functional capacity such as standing up and walking function. They usually base their assessment on a home visit, in addition to HMO diagnoses and extraneous factors such as level of income. The final purpose of the National Social Security is to determine eligibility for caregiver hours. The scale ranges from 1 (corresponding to the lowest number of caregiver hours) to 6 (corresponding to the maximum number of caregiver hours). For this paper, the scale was divided into four categories: 0 (no benefit), 1–2, 3–4, and 5–6 (the highest benefits).

### Outcome variable

A composite outcome of all-cause hospitalization or mortality. Hospitalization was identified using invoices submitted to the HMO by the hospitals. These invoices were submitted with a delay of up to two months, so they were collected in March 2023. Mortality was measured using dates of death in the population registry of Israel’s Interior Ministry.

### Statistical methods

Categorical variables were presented as numbers and percentages; the comparison between groups was performed using the chi-square test. According to abnormal distribution determined with the Shapiro-Wilk test, continuous variables were presented as median and inter-quartile; and the groups were compared using the Independent-Samples Mann-Whitney *U*-Test (for 2 groups) and the Independent-Samples Kruskal-Wallis Test (for more than 2 groups).

Time to hospitalization or mortality within 1 year was created with the Kaplan–Meier method and compared using the log-rank test to test the difference between the frailty groups (fit, mild frailty, moderate frailty, severe frailty) and post-hoc pairwise comparisons. Hazard ratios (HRs) at 1 year were estimated for the outcome of hospitalization or mortality, using multivariable hierarchical Cox proportional hazards regression, with the MEFI as the independent variable and age groups, gender, SES and CCI as covariates. We assessed for discrimination, using receiver operating characteristic (ROC) curves to estimate areas under the curve (AUC). As a sensitivity analysis, we performed the models after removing patients with missing data, and we stratified the analysis by age groups.

Data were analyzed using IBM SPSS statistics software (Version 28.0, for Windows; SPSS Inc., Chicago, IL, USA). All statistical tests were two-sided, and *p*-values lower than 0.05 were considered to be statistically significant.
